# *In Vivo* Effects of Quercetin in Association with Moderate Exercise Training in Improving Streptozotocin-Induced Aortic Tissue Injuries

**DOI:** 10.3390/molecules201219802

**Published:** 2015-12-04

**Authors:** Irina C. Chis, Andrei Coseriu, Ramona Simedrea, Adrian Oros, Andras L. Nagy, Simona Clichici

**Affiliations:** 1 Department of Physiology, “Iuliu Hatieganu” University of Medicine and Pharmacy, Number 1–3, Clinicilor Street, RO-400023 Cluj-Napoca, Romania; ramona.simedrea@yahoo.com (R.S.); sclichici@umfcluj.ro (S.C.); 2 Department of Physiology, “Iuliu Haţieganu” University of Medicine and Pharmacy, Number 8, Victor Babes Street, RO-400012 Cluj-Napoca, Romania; coseriuandrei@yahoo.com; 3 Department of Veterinary Toxicology, University of Agricultural Sciences and Veterinary Medicine, Number 3–5, Mănăştur Street, RO-400372 Cluj-Napoca, Romania; orosnadrian@yahoo.com (A.O.); nagyandras26@gmail.com (A.L.N.)

**Keywords:** endothelial dysfunction, diabetes mellitus, quercetin, exercise training

## Abstract

Background: Diabetes mellitus (DM) is a chronic endocrine-metabolic disorder associated with endothelial dysfunction. Hyperglycemia, dyslipidemia and abnormal nitric oxide-mediated vasodilatation are the major causal factors in the development of endothelial dysfunction in DM. The prevention of endothelial dysfunction may be a first target against the appearance of atherosclerosis and cardiovascular diseases. We have investigated the synergistic protective effects of quercetin administration and moderate exercise training on thoracic aorta injuries induced by diabetes. Methods: Diabetic rats that performed exercise training were subjected to a swimming training program (1 h/day, 5 days/week, 4 weeks). The diabetic rats received quercetin (30 mg/kg body weight/day) for 4 weeks. At the end of the study, the thoracic aorta was isolated and divided into two parts; one part was immersed in 10% formalin for histopathological evaluations and the other was frozen for the assessment of oxidative stress markers (malondialdehyde, MDA and protein carbonyls groups, PC), the activity of antioxidant enzymes (superoxide dismutase, SOD and catalase, CAT), nitrite plus nitrate (NOx) production and inducible nitric oxide synthase (iNOS) protein expression. Results: Diabetic rats showed significantly increased MDA and PC levels, NOx production and iNOS expression and a reduction of SOD and CAT activity in aortic tissues. A decrease in the levels of oxidative stress markers, NOx production and iNOS expression associated with elevated activity of antioxidant enzymes in the aortic tissue were observed in quercetin-treated diabetic trained rats. Conclusions: These findings suggest that quercetin administration in association with moderate exercise training reduces vascular complications and tissue injuries induced by diabetes in rat aorta by decreasing oxidative stress and restoring NO bioavailability.

## 1. Introduction

Diabetes mellitus (DM) is a chronic endocrine-metabolic disorder considered to be an important risk factor for the development of cardiovascular complications and vascular diseases, which increase the morbidity and mortality in patients with diabetes [1,2,3,4]. In diabetes both conduit arteries and resistance arteries such as the aorta are affected and the impairment of endothelial function underlies the apparition of the micro- and macro-vascular complications of diabetes [4,5]. Under physiological conditions, the entire vascular endothelium prevents the apparition of atherosclerosis by modulating the vasomotor tone and platelet activity. Inflammation and endothelial dysfunction play an important role in the development of atherosclerosis in DM [6]. Hyperglycemia, high triglyceride and low-density lipoprotein cholesterol levels, inflammation, oxidative and nitrosative stress are involved in the development of the endothelial dysfunction in diabetes [7,8,9,10].

Endothelial dysfunction includes altered anticoagulant and anti-inflammatory properties of the endothelium, dysregulation of vascular remodelling and impaired endothelium-dependent vasodilatation caused by the loss of nitric oxide (NO) bioactivity in the vessel wall [2,4,7,10].

Hyperglycemia determines endothelial dysfunction through multiple pathways such as the activation of nuclear factor (NF)-κB, which increases the expression of inducible nitric oxide synthase (iNOS) and increased generation of NO, decreased activity of endothelial nitric oxide synthase (eNOS) and enhanced mitochondrial production of the superoxide anion radicals, increased degradation of NO through its reaction with superoxide and formation of peroxynitrite [11,12,13,14]. Inducible nitric oxide synthase is a marker of vascular inflammation and its gene expression is controlled by NF-κB. NO produced in excess under iNOS action exerts pathological effects through its rapid interaction with superoxide anion radicals to form peroxynitrite, a very strong oxidant molecule which exerts toxic effects [15].

Recent studies indicate the favorable effects of natural antioxidant administration on DM to prevent the cardiovascular complications of diabetes [16,17,18,19,20]. Quercetin is a phytochemical pertaining to the flavonoid family present in citrus fruits, berries, red grapes, red wine, broccoli, tea, flowers, *etc.* [16]. Quercetin exerts multiple effects—antioxidant, antiatherogenic, antihypertensive, anti-inflammatory, angiogenesis and carcinogenesis inhibory—and recent research has shown hypoglycemic effects in DM [16,17,18,19,20].

Exercise training plays an important role in diabetes therapy. It has beneficial effects in the prevention and delay of the onset of diabetes, increase of insulin sensitivity and improvement in glucose metabolism. Recent experimental research has reported the effect of exercise training in restoring the endothelial function at diabetics by inhibiting inflammation and oxidative/nitrosative stress and by restoring NO bioavailability in vessels wall [21,22,23,24].

The aim of this study was to evaluate the effects of quercetin in association with moderate exercise training on a streptozotocin-induced experimental model of DM type 1 and aortic injuries. We hypothesized that quercetin and exercise training might improve the endothelial function and reduce aortic histopathological damage induced by streptozotocin-induced diabetes by attenuating fasting blood glucose levels and by suppressing oxidative and nitrosative stress.

## 2. Results

### 2.1. Blood Glucose and Animal Body Weights in the Experimental Groups

The fasting blood glucose (FBG) levels and body weights of all experimental rats were measured 7 days after the administration of streptozotocin (STZ) (initial FBG) and at the end of the experiment (final FBG) (Table 1). The FBG levels were found to be significantly increased (*p* < 0.0001) in the diabetic groups (DS, DE, DSQ and DEQ) compared to the sedentary untreated control rats (CS group). The moderate exercise training for 4 weeks significantly decreased the FBG levels (*p* < 0.05) in the DE group. Treatment of diabetic rats with quercetin (DSQ group) lowered the FBG significantly (*p* < 0.0001) and quercetin administration in association with moderate exercise training (DEQ group) had cumulative effects in significantly reducing the FBG levels in diabetic rats (DEQ group).

The body weight (BW) significantly decreased (*p* < 0.05) in diabetic rats. The diabetic rats (DE group) that underwent swimming training showed insignificant changes in BW when compared to the sedentary untreated diabetic rats (DS group). Treatment of diabetic rats with quercetin (DSQ group) and quercetin treatment in association with moderate exercise training in diabetic rats will not cause significant differences (*p* > 0.05) between the final and initial BW.

**Table 1:** The effect of quercetin and moderate exercise training on fasting blood glucose, body weight, fasting plasma total cholesterol and triglyceride levels in experimental groups.

Parameters	CS	CE	CSQ	CEQ	DS	DE	DSQ	DEQ
Initial FBG (mg/dL)	70.0 ± 7.4	68.5 ± 4.9	69.0 ± 7.7	67.6 ± 4.9	532.4 ± 65.9 ^aaa^	547.1 ± 71.3 ^aaa^	568.1 ± 48.7 ^aaa^	560.4 ± 57.4 ^aaa^
Final FBG (mg/dL)	77.6 ± 6.1	78.6 ± 5.9	72.6 ± 4.8	71.6 ± 4.6	546.5 ± 26.0 ^aaa^	447.0 ± 36.1 ^b^	386.2 ± 72.7 ^bbb^	302.4 ± 27.9 ^bbb^
Initial body weight (g)	237.3 ± 18.6	239.3 ± 16.5	237.4 ± 15.9	233.6 ± 18.7	251.4 ± 18.7	238.1 ± 21.3	237.4 ± 15.9	236.6 ± 16.1
Final body weight (g)	242.1 ± 18.5	233.2 ± 18.4	243.4 ± 13.9	234.7 ± 19.0	216.3 ± 8.9 ^a^	227.2 ± 12.4	232.4 ± 15.7	234.6 ± 15.8
Total-Chol (mg/dL)	78.1 ± 6.1	73.2 ± 4.3	75.3 ± 5.2	73.1 ± 4.1	203.3 ± 14.1 ^aaa^	157.9 ± 5.6 ^bbb^	135.9 ± 27.4 ^bbb^	122.4 ± 22.5 ^bbb^
TG (mg/dL)	86.1 ± 5.4	83.1 ± 5.6	81.2 ± 11.7	79.6 ± 16.0	199.2 ± 13.9 ^aaa^	132.1 ± 12.5 ^bbb^	123.3 ± 18.2 ^bbb^	109.7 ± 11.7 ^bbb^

CS = control + sedentary, CE = control + exercise, CSQ = control + sedentary + quercetin, CEQ = control + exercise + quercetin, DS = diabetes + sedentary, DE = diabetes + exercise, DSQ = diabetes + sedentary + quercetin, DEQ = diabetes + exercise + quercetin. Results are mean ± SD of 10 rats per each group. Statistically significant differences are indicated by the symbols: ^a^ *p* < 0.05, ^aaa^ *p* < 0.0001 *vs.* CS group; ^b^ *p* < 0.05, ^bbb^ *p* < 0.0001 *vs.* DS group.

### 2.2. Plasma Total Cholesterol and Triglyceride Levels in Experimental Groups

Compared to sedentary untreated control rats (CS group), the diabetic rats showed significantly higher fasting plasma total cholesterol (Total-Chol) and triglycerides (TG) values. The diabetic rats that underwent swimming training showed significantly decreased Total-Chol (*p* < 0.0001) and TG values compared with the sedentary untreated diabetic rats (DS group). quercetin administration to diabetic rats (DSQ group) significantly decreased Total-Chol (*p* < 0.0001) and TG values. The treatment with quercetin in association with moderate exercise training in diabetic rats (DEQ group) prevented the above changes in diabetic rats and improved towards normal levels (Table 1).

### 2.3. Vascular Oxidative Stress in Experimental Groups

**Figure 1:**
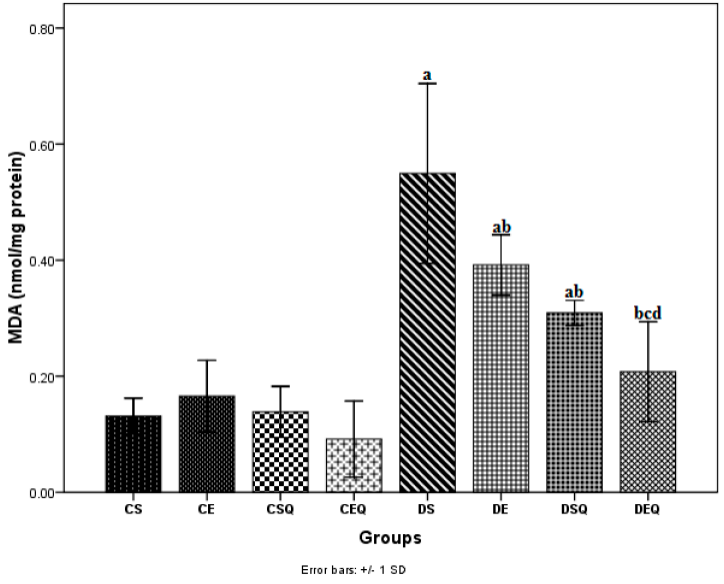
The effects of quercetin and moderate exercise training on lipid peroxidation (MDA) (nmol/mg protein) levels in the thoracic aortic tissue homogenates of control and diabetic rats. Results are the means ± SD for ten animals in each group in the CS (non-diabetic sedentary untreated control rats), CE (non-diabetic trained untreated control rats), CSQ: (non-diabetic sedentary control rats treated with quercetin), CEQ (non-diabetic trained control rats treated with quercetin), DS (diabetic sedentary control rats), DE (diabetic trained untreated control rats), DSQ (diabetic sedentary rats treated with quercetin), DEQ (diabetic trained rats treated with quercetin) groups. Statistically significant differences are indicated by the symbols: ^a^ *p* < 0.05 *vs.* CS group, ^b^ *p* < 0.05 *vs.* DS group, ^c^ *p* < 0.05 *vs.* DE group and ^d^ *p* < 0.05 *vs.* DSQ group.

The measurement of MDA level has been used as indicator of lipid peroxidation in the thoracic aortic tissue. The MDA level in aortic tissue was significantly higher (*p* < 0.05) after the induction of diabetes (DS group). Diabetic rats, when subjected to moderate exercise training (DE group), exhibited significantly decreased MDA levels (*p* < 0.05) in aortic tissue. The treatment of diabetic rats with quercetin (DSQ group) significantly decreased MDA levels (*p* < 0.05) in aortic tissue. The treatment with quercetin in association with moderate exercise training in diabetic rats (DEQ group) significantly reduced MDA levels (*p* < 0.05) in aortic tissue as compared to the control diabetic rats (DS, DE and DSQ groups) (Figure 1).

**Figure 2:**
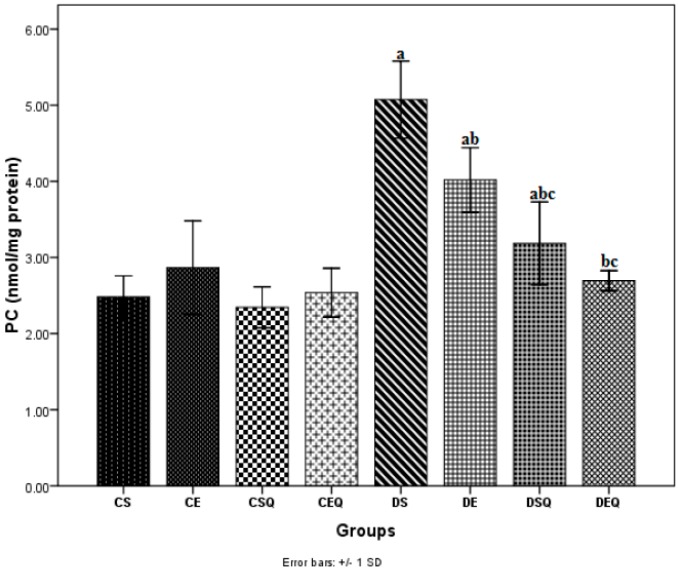
The effects of quercetin and moderate exercise training on protein carbonyl groups (PC) (nmol/mg protein) in the thoracic aortic tissue homogenates of control and diabetic rats. Results are the means ± SD for ten animals each group defined as in the caption of Figure 1. Statistically significant differences are indicated by the symbols: ^a^ *p* < 0.05 *vs.* CS group, ^b^ *p* < 0.05 *vs.* DS group and ^c^ *p* < 0.05 *vs.* DE group.

The effect of hyperglycemia on protein oxidation was measured by determining protein carbonyl (PC) groups in thoracic aortic tissue homogenate. PC levels in aortic tissue significantly increased (*p* < 0.05) after the induction of diabetes (DS group). The diabetic rats subjected to moderate exercise training (DE group) exhibited significantly decreased PC levels (*p* < 0.05) in aortic tissue. The diabetic rats treated with quercetin (DSQ group) showed significantly decreased PC levels (*p* < 0.05) in aortic tissue. quercetin administration in association with moderate exercise training in diabetic rats (DEQ group) significantly decreased PC levels (*p* < 0.05) in aortic tissue as compared to the control diabetic rats (DS and DE groups) (Figure 2).

**Figure 3:**
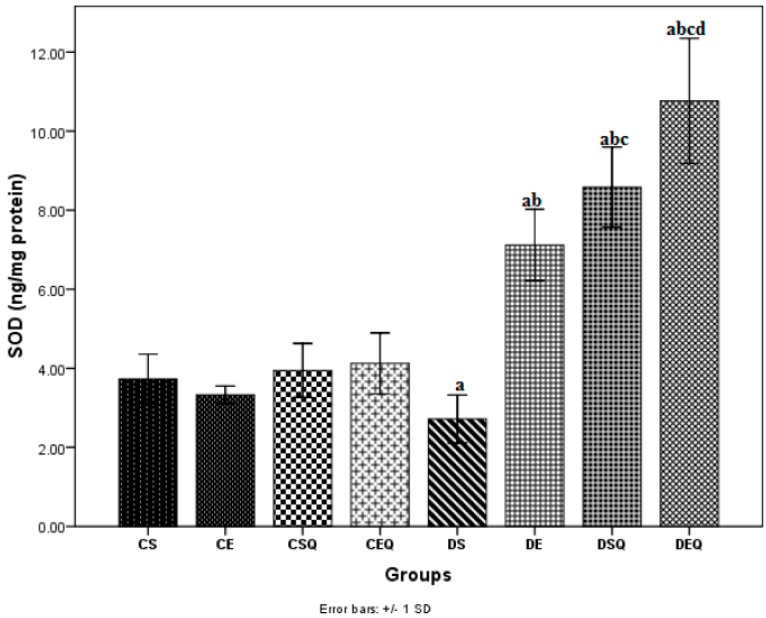
The effects of quercetin and moderate exercise training on the level of superoxide dismutase (SOD) (ng/mg protein) activity in the thoracic aortic tissue homogenates of control and diabetic rats. Results are the means ± SD for ten animals each group, defined as in the caption of Figure 1. Statistically significant differences are indicated by the symbols: ^a^ *p* < 0.05 *vs.* CS group, ^b^ *p* < 0.05 *vs.* DS group, ^c^ *p* < 0.05 *vs.* DE group and ^d^ *p* < 0.05 *vs.* DSQ group.

**Figure 4:**
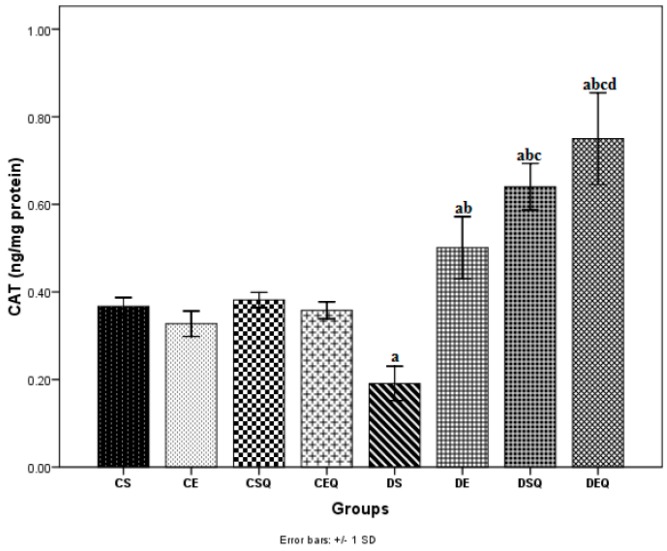
The effects of quercetin and moderate exercise training on the level of catalase (CAT) (ng/mg protein) activity in the thoracic aortic tissue homogenates of control and diabetic rats. Results are the means ± SD for ten animals each group, defined as in the caption of Figure 1. Statistically significant differences are indicated by the symbols: ^a^ *p* < 0.05 *vs.* CS group, ^b^ *p* < 0.05 *vs.* DS group, ^c^ *p* < 0.05 *vs.* DE group and ^d^ *p* < 0.05 *vs.* DSQ group.

In diabetic sedentary rats (DS group), the activities of SOD (Figure 3) and CAT (Figure 4) were significantly decreased (*p* < 0.05) in the aorta as compared to the CS group. Trained untreated diabetic control rats (DE group) exhibited a significant increase (*p* < 0.05) in the activities of these antioxidant enzymes in the aorta as compared to the DS group. The diabetic sedentary rats treated with quercetin (DSQ group) also exhibited a significant increase (*p* < 0.05) in the activities of these antioxidant enzymes in the aorta as compared to diabetic control rats (DS and DE groups). Diabetic rats treated with quercetin and subjected to moderate exercise training (DEQ group) exhibited a significantly increased (*p* < 0.05) SOD (Figure 3) and CAT (Figure 4) activity in the aorta as compared to the diabetic control rats (DS, DE and DSQ groups). Quercetin administration and the moderate exercise training had cumulative effects (*p* < 0.05) on the increase of antioxidant enzymes (SOD and CAT) in the aorta of diabetic rats.

### 2.4. Changes in Aortic Tissue Nitrite Levels and iNOS Production in Experimental Groups

**Figure 5:**
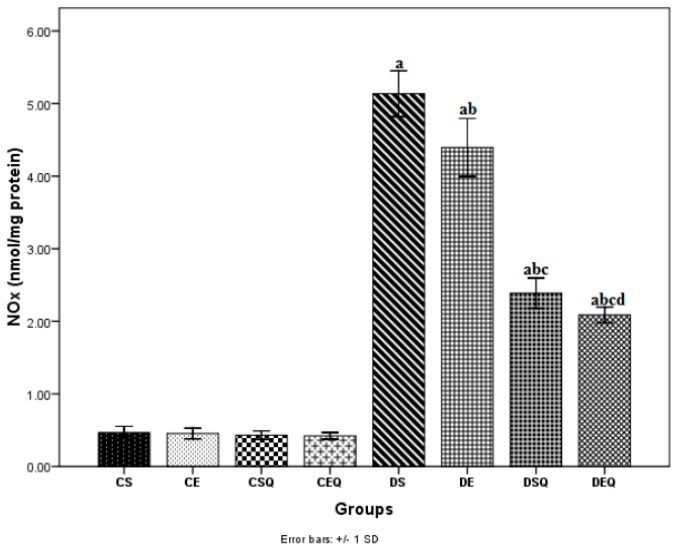
The effects of quercetin and moderate exercise training on the level of nitrite plus nitrate production (NOx) (nmol/mg protein) in the thoracic aortic tissue homogenates of control and diabetic rats. Results are the means ± SD for ten animals each group, defined as in the caption of Figure 1. Statistically significant differences are indicated by the symbols: ^a^ *p* < 0.05 *vs.* CS group, ^b^ *p* < 0.05 *vs.* DS group, ^c^ *p* < 0.05 *vs.* DE group and ^d^ *p* < 0.05 *vs.* DSQ group.

Nitrites (NOx) (biomarker for NO production) levels were increased (*p* < 0.05) as a result of diabetes (DS group) in the aortic tissue and significantly decreased (*p* < 0.05) with moderate exercise training (DE group) and respectively after quercetin administration (DSQ group). quercetin administration and moderate exercise training exerted cumulative effects (*p* < 0.05) in reducing nitrites levels in diabetic rats (DEQ group) (Figure 5).

**Figure 6:**
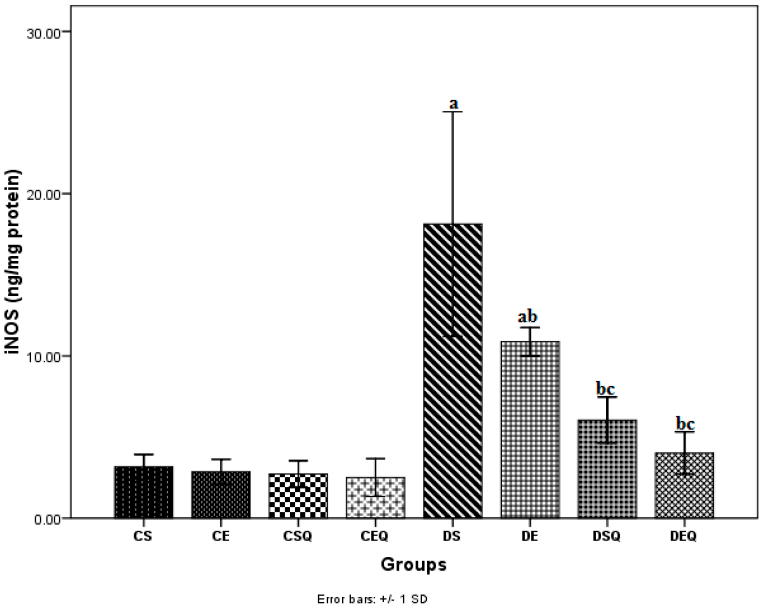
The effects of quercetin and moderate exercise training on the level of inducible nitric oxide synthase (iNOS) (ng/mg protein) in the thoracic aortic tissue homogenates of control and diabetic rats. Results are the means ± SD for ten animals each group, defined as in the caption of Figure 1. Statistically significant differences are indicated by the symbols: ^a^ *p* < 0.05 *vs.* CS group, ^b^ *p* < 0.05 *vs.* DS group and ^c^ *p* < 0.05 *vs.* DE group.

The vascular NO bioavailability in diabetes mellitus has been assessed by determining inducible nitric oxide synthase (iNOS) levels in thoracic aortic tissue (Figure 6). Levels of iNOS in aortic tissue significantly increased (*p* < 0.05) after the induction of diabetes (DS group). The diabetic rats subjected to moderate exercise training (DE group) exhibited significantly decreased iNOS levels (*p* < 0.05) in aortic tissue as compared to the diabetic control rats (DS group). The diabetic sedentary rats treated with quercetin (DSQ group) and the diabetic trained rats treated with quercetin showed significantly decreased iNOS levels (*p* < 0.05) in aortic tissue as compared to the diabetic control rats (DS and DE groups).

### 2.5. Histopathological Findings in the Thoracic Aorta

**Figure 7:**
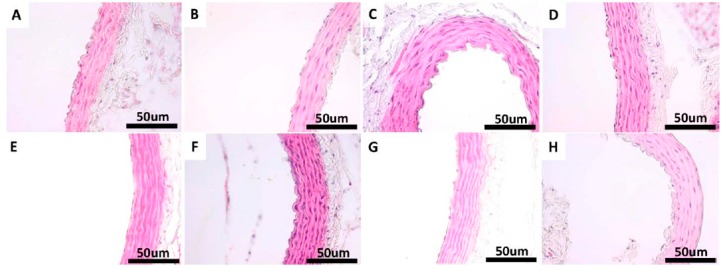
Morphology of the aorta of rats in: CS group: non-diabetic sedentary untreated control rats (**A**); CE group: non-diabetic trained untreated control rats (**B**); CSQ group: non-diabetic sedentary control rats treated with quercetin (**C**); CEQ group: non-diabetic trained control rats treated with quercetin (**D**); DS group: diabetic sedentary control rats (**E**); DE group: diabetic trained untreated control rats (**F**) and DSQ group: diabetic sedentary rats treated with quercetin (**G**); DEQ group; diabetic trained rats treated with quercetin (**H**). H & E stain original magnification of 40×. Scale bar = 50 μm.

**Figure 8:**
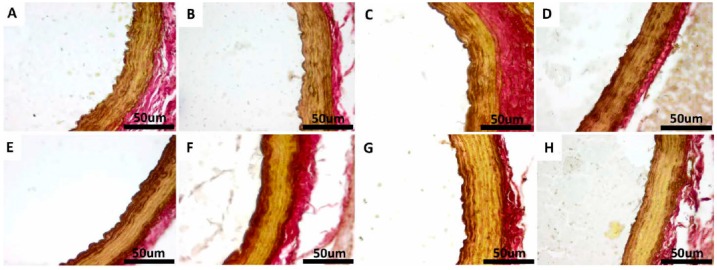
Transverse sections of the aorta stained with Verhoeff-Van Gienson (VVG) stain method for elastic fibers in: CS group: non-diabetic sedentary untreated control rats (**A**); CE group: non-diabetic trained untreated control rats (**B**); CSQ group: non-diabetic sedentary control rats treated with quercetin (**C**); CEQ group: non-diabetic trained control rats treated with quercetin (**D**); DS group: diabetic sedentary control rats (**E**); DE group: diabetic trained untreated control rats (**F**) and DSQ group: diabetic sedentary rats treated with quercetin (**G**); DEQ group; diabetic trained rats treated with quercetin (**H**). VVG stain, original magnification of 40×. Scale bar = 50 μm.

**Figure 9:**
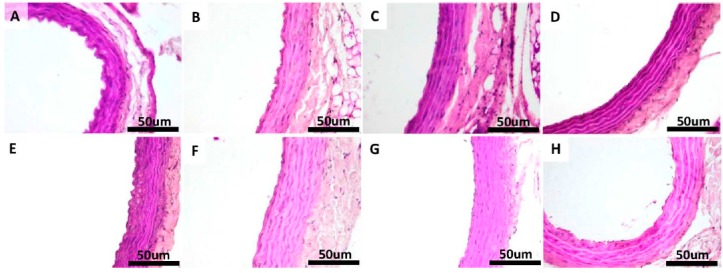
Transverse sections of the aorta stained with Periodic acid-Schiff (PAS) method in: CS group: non-diabetic sedentary untreated control rats (**A**); CE group: non-diabetic trained untreated control rats (**B**); CSQ group: non-diabetic sedentary control rats treated with quercetin (**C**); CEQ group: non-diabetic trained control rats treated with quercetin (**D**); DS group: diabetic sedentary control rats (**E**); DE group: diabetic trained untreated control rats (**F**) and DSQ group: diabetic sedentary rats treated with quercetin (**G**); DEQ group; diabetic trained rats treated with quercetin (**H**). PAS stain, original magnification of 40×.

Under H & E, VVG and PAS staining, there was no histological difference in the examined sections of thoracic aorta in the non-diabetic control groups (CS, CE, CSQ and CEQ) (Figure 7, Figure 8 and Figure 9).

In H & E staining of the aorta no lipid deposits or vacuolization of the endothelial cells were observed in any of the sections taken in study for the non-diabetic control groups (Figure 7A–D). Injury in the aortic tissue of diabetic rats (DS group) was more severe compared with non-diabetic control groups, consisting in lipid deposition in tunica intimae and tunica media and an increased thickening of media (Figure 7E). In the diabetic rats, which were subjected to moderate exercise training (DE group) and receiving quercetin (DSQ group), the severity of histopathological alterations was relatively less than that of diabetic rats (DS group) (Figure 7F–G). Lipid depositions in tunica intimae and tunica media were attenuated in rats belonging to DEQ group (Figure 7H).

Non-diabetic control groups (CS, CE, CSQ and CEQ) showed normal morphology in VVG staining of the aorta and normal distribution of the elastic fibers in all the sections taken in study (Figure 8A–D). Under VVG staining, the disruption and the reduced quantity of elastic fibers in the tunica media layer were observed in the diabetic rats (DS group) compared with non-diabetic control groups (Figure 8E). The arrangement of the elastic fibers in the DE, DSQ and DEQ groups showed less disruption compared with the DS group (Figure 8F–H).

Under PAS staining, there were no histological modifications of the aorta in non-diabetic control groups (CS, CE, CSQ and CEQ) and there was normal distribution of glycogen in all the sections taken in study (Figure 9A–D). Diabetic groups (DS, DE and DSQ groups) (Figure 9E–G) showed a marked decrease in stain intensity, with a mild increase in DEQ group (Figure 9H).

### 2.6. Morphological Findings in the Thoracic Aorta

Non-diabetic control groups (CS, CE, CSQ and CEQ groups) showed no significant changes in the thickness of TM and thickness of the aortic wall. The thickness of TM of the aorta was statistically increased in the DS group, when compared with the non-diabetic control groups (*p* < 0.05). In DEQ group, the thickness of TM of the aorta was decreased significantly (*p* < 0.05) compared with the DS group. However, the thickness of aortic wall significantly increased (*p* < 0.05) after STZ-induced diabetes (DS group) and significantly decreased after quercetin treatment in association with moderate exercise training (DEQ group) (Table 2).

**Table 2:** Measurements of the tunica media (TM) thickness of the aortic wall and aortic wall in experimental groups.

Rat Group	Thickness of the TM (μm)	Thickness of the Aortic Wall (μm)
CS	3.8 ± 0.48	51.3 ± 5.42
CE	4.22 ± 1.71	53.69 ± 1.56
CSQ	4.85 ± 0.73	48.17 ± 2.65
CEQ	4.91 ± 0.67	51.06 ± 2.23
DS	4.73 ± 1.49 ^a^	81.77 ± 4.91 ^a^
DE	4.66 ± 0.95	64.15 ± 1.59 ^b^
DSQ	4.28 ± 0.26	43.15 ± 2.97 ^b^
DEQ	2.78 ± 0.56 ^b^	30.91 ± 1.19 ^b^

CS = control + sedentary, CE = control + exercise, CSQ = control + sedentary + quercetin, CEQ = control+ exercise + quercetin, DS = diabetes + sedentary, DE = diabetes + exercise, DSQ = diabetes + sedentary + quercetin, DEQ = diabetes + exercise + quercetin. Results are mean ± SD of 10 rats per each group. Statistically significant differences are indicated by the symbols: ^a^ significant difference (*p* < 0.05) *vs.* CS group; ^b^ significant difference (*p* < 0.05) *vs.* DS group.

## 3. Discussion

Streptozotocin (STZ)-induced diabetes in rats is an accepted experimental model of DM type 1 [25] and STZ-induced vascular complications [26]. Streptozotocin is an antibiotic and chemotherapeutic produced by *Streptomyces achromogenes* which induces an inflammatory reaction of the pancreatic β-cells resulting in an absolute insulin deficiency. Cytotoxic actions in the pancreas and hyperglycemia induced by STZ are associated with the increase of oxidative stress responsible for oxidative damage and vascular dysfunction [26,27,28].

In our present study we induced type 1 diabetes in rats with STZ and we investigated the effect of quercetin treatment and moderate exercise training on diabetic-induced aortic tissue injuries, as a result of increasing oxidative and nitrosative stress levels [25,26,27,28]. After STZ administration, fasting blood glucose (FBG) levels were significantly increased in the diabetic rats. According to the findings of the present study, the administration of quercetin in dose of 30 mg/kg daily for four weeks could significantly reduce FBG levels in type 1 diabetic rats, which is in accordance with other recent studies that have revealed the hypoglycemic effect of quercetin in diabetes [19,29,30,31]. Recent work on mechanisms through which quercetin reduces the blood glucose level have explained the decrease of glycemia by: the α-glycosidase inhibitory activity *in vitro*; the increase of the hexokinase activity; the decrease in the glucose-6-phosphatase and fructo-bisphosphatase activities; the increased expression of GLUT 4 (insulin-dependent glucose transporter) via mRNA expression and translocation to the plasma membrane [30]. Diabetic rats subjected to moderate exercise training for four weeks in our study showed significantly decreased levels of FBG when compared to the sedentary diabetic rats. Our results, consistent with recent studies, showed that moderate swimming training decreased glycemia in diabetic rats [32,33]. In our research, quercetin administration in association with moderate exercise training for four weeks in diabetic rats (DEQ group) exerted cumulative effects in decreasing the FBG levels in diabetic rats. We interpreted these results in accordance with recent research, as being a consequence of protection the β-cells of Langerhans islets and to increase insulin release and the GLUT-4 expression, thus increasing the intracellular glucose transport by quercetin in association with moderate exercise training [24,29,30,32,34].

Streptozotocin-induced diabetic rats showed clinical signs that were specific to severe DM: body weight loss, polyuria, polydipsia and polyphagia associated with hyperglycemia [27,28]. The body weight of diabetic sedentary rats (DS group) in the present study was very low and the training protocol restored this body weight reduction caused by the diabetic state. Quercetin administration in diabetic rats had no effect on diabetes-induced body weight decrease but quercetin administration in association with moderate swimming training for four weeks prevented body weight reduction caused by the diabetic condition.

Cardiovascular diseases (CVD) which complicate DM are associated with increased triglycerides and decreased high-density lipoprotein (HDL)-cholesterol concentrations. Lowering cholesterol and triglycerides reduces the risk for CVD in patients with DM. quercetin may contribute to prevention or improvement of cardiovascular complications by restoring the lipid profile in DM. In STZ-induced diabetic rats, the plasma levels of total cholesterol and triglyceride were all significantly increased. These increased plasma levels were diminished after the quercetin-treatment and moderate exercise training. The present results suggest that the altered endothelial homeostasis which occurs in established diabetes is reversed by the quercetin administration in association with moderate exercise training, and that this effect may be achieved, at least partially, through a decrease of FBG, total cholesterol and triglyceride levels. These results are in accordance with recent research, which has proven the hypotriglyceridemic and hypocholesterolemic effects of quercetin and respectively of the moderate exercise training [19,21,35].

Hyperglycemia, dyslipidemia, insulin resistance and abnormal nitric oxide (NO) mediated vasodilatation are the major causal factors in the development of endothelial dysfunction in DM. In response to hyperglycemia, diabetic endothelium produces an increase in reactive oxygen species (ROS), mainly superoxide anion radicals, playing a key role in the pathogenesis of vascular complications [2,4,7,10]. In this study, MDA and PC levels were found significantly increased while the antioxidant activities were lower in the aortic tissue, which proves the presence of oxidative stress in the aorta of STZ-induced diabetic rats.

Quercetin reduced MDA and PC levels and increased antioxidant SOD and CAT activities in the aorta, suggesting that quercetin behaves as a strong antioxidant in this animal model. Quercetin was reported to be a strong antioxidant by increasing endogen antioxidant activities and by directly scavenging free radicals [16,36,37,38]. Recent studies proved that quercetin is a strong antioxidant which exerts its vasodilator endothelium-dependent effects by protecting the bioavailability of NO and endothelial function and antiatherogenic effects in the presence of inflammatory lesions, triggered by oxidative stress [30,39].

Physical activity is recommended in the management of human diabetes [40] because it decreases body weight and improves glycemic control and plasma lipids. Loss of body weight has beneficial effects on cardiovascular complications. By reducing hyperglycemia, inflammation, oxidative and nitrosative stress, exercise training improves endothelial function and vasodilatation [21,22,23,24]. In our study, after four weeks of moderate exercise training, MDA and PC levels significantly decreased and antioxidant activities significantly increased in the aortic tissue of diabetic rats. These results demonstrate the beneficial antioxidant effects of moderate exercise training in diabetes which may restore the endothelial function.

Hyperglycemia also favours, through the activation of nuclear factor (NF)-κB, an increased expression of inducible nitric oxide synthase (iNOS), which is accompanied by increased generation of NO [41]. Relatively low physiological concentrations of NO produced under endothelial nitric oxid synthase (eNOS) action are responsible for its physiological effects (the relaxation of blood vessels, maintenance of blood pressure, platelet antiaggregant, *etc.*). Nitric oxide produced in increased quantities by iNOS exerts pro-oxidant pathological effects. A marker of vascular inflammation in diabetes is the production of iNOS, which increases NO production and contributes to the endothelial dysfunction. NO can react with superoxide anion radical to form the strong oxidant peroxynitrite, which in turn can increase lipid peroxidation, protein nitration, and low-density lipoprotein (LDL) oxidation, affecting many signal transduction pathways and thus has a pro-atherogenic role [11,12,13,14,15,42]. Recent experimental evidence supports the idea of complex roles for NO, ROS and peroxynitrite in the development of early diabetes tissue injury before the evolution of late complications [43,44]. One of the mechanisms by which quercetin exerts its anti-inflammatory effects consists in inhibiting the production of tumor necrosis factor alpha (TNFα) and iNOS in macrophages [36,37]. In the present study, the nitrites and iNOS levels in aortic tissue significantly increased after the induction of diabetes. These data clearly prove the presence of inflammation and nitrosative stress in aorta after the induction of diabetes. In our study, quercetin decreased significantly the nitrite and iNOS levels in diabetic rats. The association of quercetin with moderate exercise training in diabetic rats reduced even more the nitrite and iNOS levels, proving the beneficial effects of this association in endothelial dysfunction induced by DM.

These results suggest that the administration of quercetin in association with moderate exercise training exerts synergistic effects in restoring the endothelial homeostasis of aorta in STZ-diabetic rats, possibly due to the significant attenuation of hyperglycemia, hypotriglyceridemia, hypo-cholesterolemia and by decreasing oxidative/nitrosative stress and preserving the structural and functional integrity of the aortic tissues.

Another major finding of our study relates to the aortic damage in STZ-induced diabetic rats, which was reduced after quercetin treatment in association with moderate exercise training. The histological findings of this study showed that the structural organization of the aorta was disrupted in STZ-induced diabetic rats. In addition, increased thickness of the tunica media and aortic wall was observed in diabetic rats (DS group). In this study, the STZ injection significantly increased the thickness of tunica media and aortic wall. In human studies, tunica media thickness of aorta increased significantly in diabetes compared with that of healthy people [45,46]. The present study showed that quercetin administration ameliorated the thickness of aortic wall and organization of elastic fibrils. On the other hand, quercetin had beneficial effects on the histological injury induced by STZ treatment. We found that quercetin in association with moderate exercise training reduced the medial thickening.

STZ-induced diabetes increased aortic tissue damage, highlighted by marked increase of oxidative and nitrosative stress associated with higher lipid deposition and thickening of blood vessel wall. The treatment with quercetin and moderate exercise training significantly reduced aortic pathological damage induced by diabetes and these effects were accompanied by increased antioxidant enzymes (SOD and CAT) activities levels and reduced MDA, PC, nitrites and iNOS levels in aortic tissue of diabetic rats. These findings suggest that treatment with quercetin and moderate exercise training restores vascular structural and functional integrity and histopathological alterations in diabetes.

## 4. Experimental Section

### 4.1. Drugs and Chemicals

Streptozotocin (STZ) and quercetin (Que) were obtained from Sigma-Aldrich Chemical Company Inc., (Gillingham, Dorset, UK). Streptozotocin was freshly dissolved in citrate sodium buffer (0.1 mol/L, pH 4.5) and maintained on ice before being used. Que was suspended in 0.5% carboxymethylcellulose (CMC) solution as a vehicle.

### 4.2. Animals

Eighty healthy Wistar albino male rats (three months old) were used in this study. The animals were divided into eight experimental groups. The rats were purchased from the Experimental Animal House of the Faculty of Medicine within “Iuliu Hatieganu” University of Medicine and Pharmacy of Cluj-Napoca, Romania. The rats’ average weight was 250–300 grams (g) at the beginning of the trials. All the animals used in the experiment were kept for ten days to acclimatize to the conditions of the Animal House Laboratory at the Physiology Department before being introduced in the study. Throughout the entire period of the experiment, all the rats were maintained in special cages, which were kept under control, artificially illuminated (12 h dark/12 h light cycle), at a temperature of 21–23 °C and at 50%–60% humidity in an animal room. The animals were given standard rat pellets diet and water *ad libitum*. All the experiments were performed according to the approved animal protocols of the Ethical Committee on Animal Welfare of “Iuliu Hatieganu” University in accordance with the Romanian Ministry of Health and complying with Guidelines in the Use of Animals in Toxicology.

### 4.3. Induction of Experimental Diabetes Mellitus by Streptozotocin

The study was carried out on an animal model with type 1 diabetes, induced by STZ. After overnight fasting (*i.e.*, the rats were deprived of food for 12 h but given free access to water), diabetes mellitus was induced in rats via a single intraperitoneal (i.p.) injection of STZ (50 mg/kg body weight) in freshly prepared citrate sodium buffer [47,48,49]. Streptozotocin injected animals were given an i.p. injection with 1 mL of 10% glucose saline, 30 min after STZ administration to prevent initial drug-induced hypoglycemic mortality [50,51].

Animals from the control groups were given only a single i.p. injection of citrate sodium buffer at the same volume used to dissolve STZ. Afterwards, 96 h after the STZ administration, diabetes was confirmed by measuring the fasting blood glucose (FBG) concentration. Rats that had a higher FBG level than 250 mg/dL (13.89 mmol/L) were included in the study as diabetic rats. One week after the STZ administration (the 8th day), the FBG level was measured and the quercetin administration and exercise training started.

The blood was drawn from the retro-orbital venous plexus of overnight fasting animals into tubes containing potassium oxalate and sodium fluoride as anticoagulant, whereas the glucose levels in the whole blood was estimated using the ACCU-CHEK Sensor System from Roche Diagnostics GmbH (Mannheim, Germany). The monitoring of FBG level was made through a quick method, using a single drop of capillary blood, sampled from the level of the tail, applied on a glucose test strip (Glucometer ACCU-CHEK Go, Roche).

### 4.4. Moderate Exercise Training Protocol

Every day, animals were transported to an experimental room where they were forced to swim. Three rats from each exercise group were placed in a cylindrical tank with a diameter and height of 60 and 100 cm, respectively a depth of 30–45 cm. To minimize stress associated with cold or hot water exposure, water temperature was monitored and maintained at 36 °C. The rats were trained to swim for over one week, for 15 min/day (5 days/week); the exercise protocol was gradually increased by 15 min/day until a swimming period of 1 h/day (1 week) was attained in order to reduce water-induced stress. After the initial training, the rats went through chronic moderate exercise for 1 h/day, 5 days/week, for 4 weeks. The animals were forced to swim until they became exhausted and remained on the surface. The exercise program was conducted essentially as described by Teixeira de Lemos *et al.* [21]. With some modifications, though. The sedentary rats were subjected to the same sampling and handling procedures as the exercise training animals, but they remained in their cages without food and water for the duration of the swimming exercise period.

### 4.5. Experimental Design

The animals were randomly divided into eight experimental groups (*n* = 10): the 1st Group (control + sedentary, CS)-non-diabetic, sedentary untreated control rats; the 2nd Group (control + exercise, CE)-non-diabetic, trained untreated control rats; the 3rd Group (control + sedentary + quercetin, CSQ)-non-diabetic, sedentary control rats treated with quercetin; the 4th Group (control + exercise + quercetin, CEQ)-non-diabetic, trained control rats treated with quercetin; the 5th Group (diabetes + sedentary, DS)-diabetic, sedentary untreated control rats; the 6th Group (diabetes + exercise, DE)-diabetic, trained untreated control rats; the 7th Group (diabetes + sedentary + quercetin, DSQ)-diabetic, sedentary rats treated with quercetin; the 8th Group (diabetes + exercise + quercetin, DEQ)-diabetic, trained rats treated with quercetin.

The quercetin was orally administered via an intragastric tube (0.6 mL/rat), in a dose of 30 mg/kg body weight once a day for 4 weeks. The rats from the groups: CS, CE, DS and DE were treated with CMC (0.6 mL/rat) administered orally once a day for 4 weeks. The quercetin and CMC administration in diabetic rats started one week after the STZ administration.

Then the animals were included in a long-term study of 35 days (5 weeks). For the entire duration of the experiment, the following features were monitored in each animal: the water consumption (daily), food consumption (weekly), body weight (BW) (weekly), mortality (daily) and diuresis (daily). The FBG level was measured in all the experimental animals at the beginning of the experiment, 96 h after the STZ administration, 7 days after the STZ administration and at the end of the experiment.

Seven days after the STZ administration (the 8th day) and at the end of the experiment (the 35th day) the levels of fasting plasma lipids (total cholesterol, Total-Chol and triglycerides, TG) were quantified using ACCU-TREND Sensor System from Roche Diagnostics GmbH. Five weeks after the STZ administration, the diabetic animals as well as the time-matched controls were killed and thoracic aortic samples were collected.

### 4.6. Sampling and Tissue Processing

After overnight food deprivation, the blood glucose of the rats was measured using a glucometer and then the animals were anesthetized with an i.p. injection of sodium pentobarbital (60 mg/rat) and sacrificed by cervical dislocation. The thoracic aorta was rapidly removed and rinsed in cold saline. The aortic tissue was divided into two parts: one part was frozen with liquid nitrogen and stored in a −80 °C refrigerator until biochemical assays and the other part was immersed in 10% formalin for histopathological evaluation.

### 4.7. Preparation of Aortic Tissue Homogenates

Thoracic aortic samples were homogenized in cold physiological saline solution (PSS) at pH 7.4 in the presence of protease inhibitor cocktail (104 mM AEBSF, 0.08 mM aprotinin, 2 mM leupeptin, 4 mM bestatin A and 1.4 mM E-64) (Sigma-Aldrich, St. Louis, MO, USA) with a Brinkman Polytron Kinematica homogenizer (Lucerne, Switzerland). Then, the homogenate was centrifuged at 10,000 rpm for 10 min at 4 °C. The supernatants were removed from the homogenates and quickly frozen at −80 °C until determination of biochemical markers.

### 4.8. Measurement of Biochemical Markers of Oxidative and Nitrosative Stress

Levels of oxidative stress in the aortic tissue homogenate were indirectly measured through the measuring of the free radical production. The following biochemical markers of the effects of free radicals on lipid peroxidation and protein carbonylation were determined.

The lipid peroxidation was measured from the aortic tissue homogenate by measuring the malondialdehyde (MDA) levels (using the fluorimetric method with 2-thiobarbituric acid described by Conti method) [52]. The MDA was spectrofluorimetrically determined in the organic phase using a synchronous technique with excitation at 534 nm and emission at 548 nm. The MDA levels were expressed as nanomole per milligram protein (nmol MDA/mg protein).

Protein carbonylation was estimated from the aortic tissue homogenate by measuring protein carbonyl (PC) group levels. The protein carbonyl derivates that are produced through the protein oxidative damage were determined using the fluorimetric method with 2,4-dinitrophenyl-hydrazine (DNPH) [53]. The readings were performed using a spectrophotometer at 355–390 nm and in order to calculate the remaining carbonyl fragments, the molar extinction coefficient with a value of 22,000/M/cm was used. The levels of the carbonyl-derivative groups were expressed as nanomole per milligram of protein (nmol/mg protein).

The activities of some antioxidant enzymes, superoxide dismutase (SOD) and catalase (CAT) in the aortic tissue homogenate were assayed. The enzyme-linked immunosorbent assay (ELISA, R & D Systems, Minneapolis, MN, USA) was performed to determine the SOD activity. The SOD activity was also expressed as units per milligram of protein (ng/mg protein).

The CAT activity was assayed using the method proposed by Pippenger *et al.* [54]. The method consists in following the change in absorbance of a solution of H_2_O_2_ 10 mM in potassium phosphate buffer 0.05 M, pH = 7.4 to 240 nm. One unit of CAT is defined as the amount of enzymes which induces reduction in the absorbance of 0.43 at 25 °C for 3 min. The CAT activity was also expressed as units per milligram of protein (U/mg protein).

The nitrosative stress markers in aortic tissue homogenate were determined by measuring the nitric oxide (NO) and inducible nitric oxide synthase (iNOS) levels. NO production was detected indirectly by measuring the degradation products of NO, the total nitrite level, after a prior conversion of nitrates into nitrites under the action of nitrate reductase. The nitrite plus nitrate (NOx) production was determined by measuring the nitrite, a stable end-product of NO metabolism using the Griess reaction (Titherage method) [55]. The supernatant was mixed with an equal volume of Griess reagent followed by spectrophotometric measurement at 543 nm. Nitrite concentrations in the aortic tissue homogenate were determined by comparison with a sodium nitrite standard curve. The NOx levels are presented as nanomole per milligram of protein (nmol/mg protein).

The aortic tissue homogenate level of iNOS was measured using commercially available ELISA kit (R & D Systems). The iNOS levels are expressed in nanogram per milligram of protein (ng/mg protein).

### 4.9. Light Microscopy Studies of the Thoracic Aorta

Samples of thoracic aorta were taken during necropsy and fixed in 10% neutral buffered formalin. The samples were embedded in paraffin and cut at 4 micrometers. The slides were stained using hematoxylin and eosin (H & E) stain, Verhoeff-Van Gienson (VVG) and Periodic acid-Schiff (PAS) stain methods. Then the slides were examined under a BX51 microscope Olympus (2 Corporate Center Drive, Melville, NY, USA). The images were taken with an Olympus UC 30 digital camera and processed by a special image acquisition and processing program (Olympus Stream Basic). Each aorta from every animal had its vessel width measured at three different points.

Smooth muscle cell width was measured by determining the space between elastic bands at three different points along the vessel. All analyses were completed on images taken at 40× magnification [29].

Verhoeff-Van Gienson elastic stain method was used to observe the distribution of elastic fibers in the aorta of animals from different experimental groups.

**Scheme 1:**
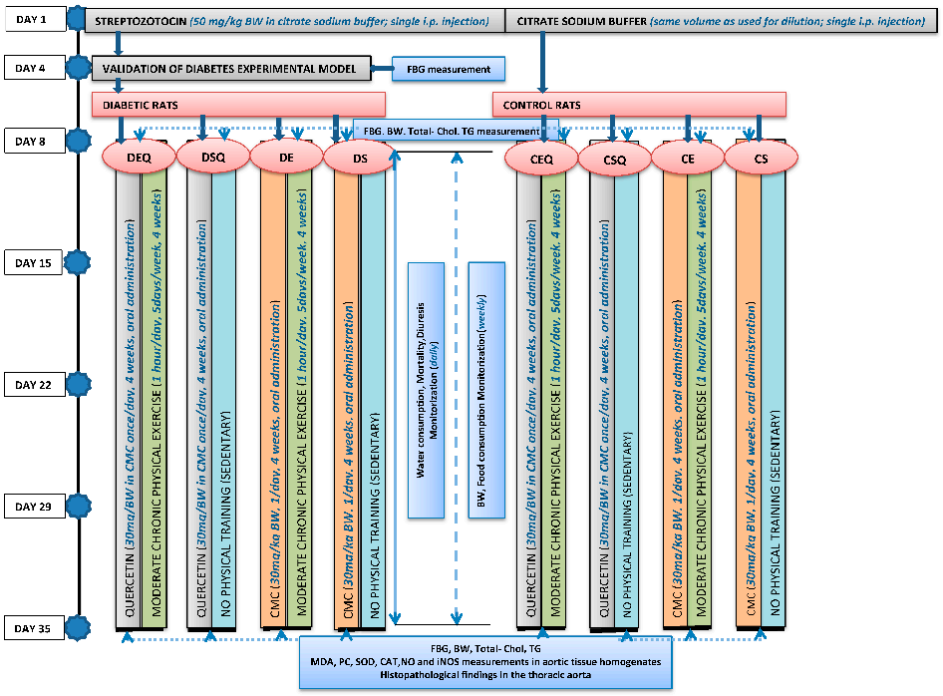
The experimental design.

Periodic acid-Schiff stain was used to evaluate the glycogen quantity and distribution within aorta wall. A graph illustrating the experimental design is shown in Scheme 1.

## 5. Statistical Analysis

The statistical analysis was performed using the SPSS software package (version 17.0, SPSS Inc., Chicago, IL, USA). The data were reported as mean ± SD. One-way analysis of variance (ANOVA) was used to compare differences between groups, and two-way ANOVA for repeated measurements, followed by Tukey’s multiple posttest comparisons, to compare the responses to quercetin and exercise. Three-way ANOVA was used to compare cumulative response to quercetin and exercise in diabetes. Differences were considered significant if *p* < 0.05.

## 6. Conclusions

In conclusion, our findings suggest that quercetin administration in association with moderate exercise training improved hyperglycemia, hypertriglyceridemia, hypercholesterolemia and antioxidant status in STZ-diabetic rats. Quercetin and moderate exercise training might improve the endothelial function and reduce the aortic histopathological damage induced by STZ-induced diabetes through attenuating fasting blood glucose level and by suppressing oxidative and nitrosative stress, thereby increasing NO bioavailability.
